# Optimizing the Adipogenic Induction Protocol Using Rosiglitazone Improves the Physiological Parameters and Differentiation Capacity of Adipose Tissue-Derived Mesenchymal Stem Cells for Horses, Sheep, Dogs, Murines, and Humans

**DOI:** 10.3390/ani13203224

**Published:** 2023-10-15

**Authors:** Manuela Heimann, Mohamed I. Elashry, Michele C. Klymiuk, Asmaa Eldaey, Sabine Wenisch, Stefan Arnhold

**Affiliations:** 1 Institute of Veterinary Anatomy, Histology and Embryology, Justus-Liebig-University of Giessen, 35392 Giessen, Germany; manuela.heimann@vetmed.uni-giessen.de (M.H.); michele.klymiuk@vetmed.uni-giessen.de (M.C.K.); stefan.arnhold@vetmed.uni-giessen.de (S.A.); 2 Clinic of Small Animals, c/o Institute of Veterinary Anatomy, Histology and Embryology, Justus-Liebig-University of Giessen, 35392 Giessen, Germany; asmaa_awad47@yahoo.com (A.E.); sabine.wenisch@vetmed.uni-giessen.de (S.W.)

**Keywords:** adipose tissue, mesenchymal stem cells, proliferation, adipogenic differentiation, apoptosis

## Abstract

**Simple Summary:**

Mesenchymal stem cell investigation has become an interesting scientific field in regenerative medicine and organ transplantation, particularly for chronic incurable disorders and massive tissue loss. Under suitable laboratory conditions, stem cells are able to renew themselves but also differentiate in bone, fat, and cartilage, which can be used to replace either damaged or lost tissue in veterinary medicine. Improper stimulation protocols cause low cell viability, death, and the failure of tissue repair. We used fat formation as a model to understand the cell specification requirements and to establish a common protocol to improve the cell activity and specification capacity for several mammalian species including humans. Thus, we replaced a widely used fat formation protocol referred to as 3-isobutyl-1-methylxantine and indomethacin with rosiglitazone. The results show that using 1–5 µM of rosiglitazone sufficiently improves the cell viability parameters, prevents cell death, and enhances fat formation compared to the traditional 3-isobutyl-1-methylxantine and indomethacin protocol. We found differences between the amount of fat formation and the level of the fat-regulating genes between species. This report provides essential information regarding the selection of a stem cell specification program and the requirement for each species for the further reproducibility and clinical targeting of obesity and metabolic disorders in veterinary medicine.

**Abstract:**

The investigation of adipose tissue-derived mesenchymal stem cells (ASCs) has received considerable interest in regenerative medicine. A nontoxic adipogenic induction protocol valid for cells of different mammalian species has not been described. This study aims to establish an adipogenic differentiation protocol suitable for horses, sheep, dogs, murines, and human cells. An optimized rosiglitazone protocol, consisting of 5% fetal calf serum in Dulbecco’s Modified Eagle’s Medium, 10 μg/mL insulin, 0.55 μg/mL transferrin, 6.8 ng sodium selenite, 1 μM dexamethasone, and 1–5 μM of rosiglitazone, is compared to the 3-isobutyl-1-methylxantine (IBMX) protocol, where rosiglitazone was replaced with 0.5 mM IBMX and 0.2 mM indomethacin. Cell viability, cytotoxicity, a morphometric analysis of the lipid, and the expression of adipogenic markers for 14 days were assessed. The data revealed that using 5 µM of rosiglitazone promotes the adipogenic differentiation capacity in horse, sheep, and dog cells compared to IBMX induction. Meanwhile, marked reductions in the cell viability and cell number with the IBMX protocol were detected, and rosiglitazone increased the cell number and lipid droplet size, prevented apoptosis, and upregulated *FABP-4* and *Leptin* expression in the cells of most of the species. Our data revealed that the rosiglitazone protocol improves the adipogenesis of ASCs, together with having less toxicity, and should be considered for cell reproducibility and clinical applications targeting obesity.

## 1. Introduction

Adult mesenchymal stem cells (MSCs)’s characterization and differentiation in vitro have been introduced as a possible therapeutic approach in regenerative medicine. Adult MSCs are able to differentiate into several lineages, including osteogenic, chondrogenic, adipogenic, tenogenic, myogenic, and neurogenic fates, by using the appropriate induction protocols [1]. The basic prospect of using these cells is to repair tissue injuries and to restore tissue degeneration when it becomes unresponsive to traditional treatment options. Despite the restricted differentiation potential, MSCs remain valuable resources of cell-based therapies in metabolic diseases, disorders of the immune system, and cancer and blood disorders [2,3,4,5]. MSCs’ transplantation has been used in conditions of severe skin burns and showed the impressive repair of the affected lesions [6]. Recently, MSC-based products have been approved in the European market for the treatment of corneal damage;; a report showed improved vision recovery following the in vitro cultivation and transplantation of either corneal endothelial cells in the presence of a p-associated kinase (ROCK) inhibitor or autologous limbal epithelial cells, reviewed by [7]. The application of MSCs for the treatment of autoimmune diseases and rheumatoid arthritis is already under clinical investigation [8]. MSCs’ induction into an adipogenic fate provides a beneficial approach for the reconstruction of soft tissue atrophy, tumor operations, lipodystrophy, and congenital anomalies [9]. Alongside applications in human medicine [10,11], investigating the differentiation capacity of MSCs also has a great impact on various disciplines of veterinary regenerative medicine, including orthopedics, ophthalmology, neurology, and dermatology in canines [12]. Furthermore, MSCs have shown effectiveness and beneficial therapeutic values in immunomodulation, digestive tract, and respiratory, dermal, and neuromuscular diseases in canines and equines, as reviewed [13]. Therefore, the characterization of MSCs and optimization of their differentiation capacity, not only for adipogenesis but also for other lineages, including osteogenesis, chondrogenesis, and tenogenesis, require further investigations. 

Adipose tissue offers a useful source for the isolation of mesenchymal stem cells (ASCs) since it has been proven that the proportion of regenerative cells in fat tissue is much higher compared to cells isolated from bone marrow (BMSCs). It was reported that a higher number of stem cells could be isolated from adipose tissue compared to a similar amount of bone marrow in equine and human cells [14,15]. The stemness properties, including self-renewal and multipotential differentiation, are higher in ASCs, and it was found that cell senescence was detected early in passage seven for BMSCs. However, ASCs could be cultivated up to passage eight without signs of senescence [16]. In addition, a study recommended the suitability of ASCs for diabetes mellitus due to their higher differentiation capacity into pancreatic beta-islet cells compared to BMSCs [17]. Furthermore, ASCs appeared to be an active alternative for improving the repair of sciatic nerve injury [18]. By considering the availability of adipose tissue in sufficient quantities for their use in regenerative medicine, it has become a routine investigation to study the differentiation performance of stem cells, including their adipogenic fate. A wide range of different protocols have been developed to induce the adipogenic differentiation of MSCs; however, the majority of protocols rely on dexamethasone (DXM), 3-isobutyl-1-methylxantine (IBMX), and insulin. Other studies have recommended indomethacin (INDO) as an additional supplement to the adipogenic differentiation cocktail [9]. Previous studies revealed that IBMX, an inhibitor of cyclic nucleotide phosphodiesterases (PDEs), increases the cellular levels of cyclic adenosine monophosphate (cAMP), as well as guanosine 3′,5′-cyclic monophosphate (cGMP), and activates cyclic nucleotide-regulated protein kinases [19]. In combination with DXM, IBMX modulates PPARγ, the master regulator of adipogenic differentiation [9]. Both DXM and IBMX act as inducers of the basic leucine zipper (C/EBPδ), which plays a role in growth and differentiation [9]. INDO is a nonselective inhibitor of cyclooxygenases (COX-1 and COX-2) belonging to nonsteroidal anti-inflammatory drugs. In combination with IBMX, DXM, and insulin, INDO promotes the adipogenic differentiation of human stem cells in vitro [20,21]. Although the current differentiation protocols are widely used, variable outcomes and discrepancies could be detected. For example, the PPARγ2 ligand rosiglitazone has been found to upregulate aP2 and adiponectin expression similarly to INDO and to increase lipid droplet formation in CH310T1/2 stem cells [22]. In addition, it was concluded that INDO improves adipogenic differentiation by upregulating the PPARγ2 and C/EBPβ expression independent of a prostaglandin modulation [22]. The compositions of the adipogenic induction media not only varied between the species but also between tissue origin of the same species [9]. It is evident that each individual substance of the differentiation protocols has dose-dependent effects on the gene expression of the differentiating cells. Additionally, the components of fetal calf serum may influence adipogenesis by themselves. Therefore, it is difficult to compare the results of experiments with different media compositions in order to define a common adipogenic differentiation protocol. A major restriction to completely understanding adipogenesis in in vitro cultivation systems is the lack of standard adipogenic differentiation protocols suitable for many, if not all, animal species. Thus, the aim of the present study was to develop a standard medium for adipogenesis that could be used for stem cells derived from various species, including horses, sheep, dogs, rats, and mice, compared with humans. 

## 2. Materials and Methods

### 2.1. Chemical and Bioethics

Adipose tissue was collected from the Institutes of Veterinary Pathology and the Clinic for Small Animals Surgery as well as the Clinic for Horses Surgery, laboratory animal house, at Justus-Liebig University of Giessen, by following standard and ethical regulations. All husbandry and experimental procedures were approved by the institutional ethics committee (Nr. V 54-19 c 20 15 h 02 GI 18/1 kTV 1/2018 and the Regierungspräsidium Gießen GI 20/10 Nr. 105/2014). Human Primary ASCs (lot Nr. 428Z005.3, 430Z013, 423Z037.1) were purchased from Promocell (Heidelberg, Germany). The study design, including hypothesis and aims, experimental setup, targeted objectives, and data analysis, is collectively illustrated in (Figure S1a).

### 2.2. Isolation of ASCs 

Subcutaneous and intra-abdominal adipose tissue samples (*n* = 3) from female (mean ± SD) 13 ± 8-year-old horses, 2–4-week-old sheep, 5 ± 3-year-old dogs, 4–8-week-old Wistar rats, 4–6-week-old C57BL/6 mice, and 41 ± 9-year-old humans were collected in previously cooled phosphate-buffered saline (PBS, Gibco, Life technologies, Darmstadt, Germany) with 1% penicillin/streptomycin (P/S, Gibco, Life technologies, Darmstadt, Germany) under sterile conditions. Adipose tissue from horses, sheep, and dogs was collected after a routine surgical operation at Giessen University’s local clinic. The fat specimens were cut into 1 mm^2^ pieces, and then were enzymatically digested with an equal amount of Dulbecco’s Modified Eagle’s Medium low glucose (DMEM-LG, Gibco, Life technologies, Darmstadt, Germany), containing 1mg/mL collagenase Type I, CLS I (Biochrom, Merck Millipore, Darmstadt, Germany) using a Tube Rotator for 1 h at 37 °C. After centrifugation at 240× *g* for 5 min at room temperature, the pellet was carefully aspirated using a serological pipette and filtered through a 70 μm cell strainer, washed with PBS, and centrifuged a second time at 240× *g* for 5 min at room temperature. The supernatant was removed, and the pellet was suspended with DMEM-LG, supplemented with 10% fetal calf serum (FCS, Capricorn, Ebsdorfergrund, Germany) and 1% P/S and incubated in a humidified atmosphere at 37 °C under 5% CO_2_. Identification and characterization of ASCs were performed by using standard applications, including PCR, flow cytometry, and immunohistochemical staining for specific stem cell markers. The list of primary and secondary antibodies used in the current study to detect ASC markers, their target species, and their cross reactivity between species are listed in (Table S1). The protocol for ASCs’ multipotency was previously described in detail by our group in horse [23], dog [24], and rat cells [25]. Cells of passages P1–P4 from all animal groups and humans were expanded and then used for all experimental procedures. 

### 2.3. Adipogenic Differentiation

ASCs were plated at a density of 1 × 10^4^ cells/cm^2^ to 24-well plates (VWR, Darmstadt, Germany), cultivated in DMEM-LG, supplemented with 10% FCS and 1% P/S, and incubated at 37 °C under 5% CO_2_. Three days after seeding, differentiation induction was carried out using various adipogenic induction media. (1) Cells processed in parallel in basal medium (BM) consisting of DMEM high glucose (DMEM-HG, Gibco, Life technologies, Darmstadt, Germany) supplemented with 5% FCS and 1% P/S served as negative control. (2) The commonly used standard protocol (IBMX) consisted of DMEM-HG supplemented with 5% FCS, ITS (10 μg/mL insulin, 0.55 μg/mL transferrin, and 6.8 ng sodium selenite) (ITS, Sigma-Aldrich, Taufkirchen, Germany), 1 μM DXM (Sigma-Aldrich, Taufkirchen, Germany), 0.5 mM 3-isobutyl-1-methylxanthine (IBMX, Sigma-Aldrich, Taufkirchen, Germany), and 0.2 mM INDO (Sigma-Aldrich, Taufkirchen, Germany). (3) Our modified adipogenic differentiation protocol consisted of 5% FCS in DMEM-HG supplemented with ITS, 1 μM DXM, and various concentrations of rosiglitazone (Sigma-Aldrich, Taufkirchen, Germany) 1 μM (Rosi 1), 5 μM (Rosi 5), and 10 μM (Rosi 10). Cells were differentiated for 7 and 14 days, with the medium changed every three to four days. Note: Rosi 10 was used only to examine the effect of a high concentration of rosiglitazone on cell viability, cell number, and cytotoxicity but not for the adipogenic differentiation of ASCs.

### 2.4. Oil Red O Staining

Adipogenic differentiated cells from all experimental groups were fixed after 7 and 14 days in 4% paraformaldehyde at RT for 20 min. Three parts 0.3% Oil Red O Stock solution (ORO, Merck, Darmstadt, Germany) in 100% isopropanol were mixed with two parts distilled water and filtered through a 0.2 µm filter after 20 min incubation. The cells were washed twice with PBS for 5 min, once with 60% isopropanol for 3 min, and stained for 20 min with the freshly filtered staining solution. Afterward, the unbound dye was washed off with 60% isopropanol until a clear solution was obtained. The dyeing remained durable by over coating with deionized water. ORO-stained cells were photographed using an inverted light microscope equipped with a *Leica MC170 HD* camera (Leica Microsystems, Wetzlar, Germany) and operated with LAS V4.4 software (Leica, Wetzlar, Germany). For semi-quantification of lipid droplets, water was removed from the cells, the bound dye was extracted with 200 µL of 100% isopropanol by mild shaking for 30 min, and the extract was measured using a Tecan Sunrise Absorbance Microplate Reader (Tecan Deutschland GmbH, Crailsheim, Germany) at an absorbance of 492 nm. For data analysis, MagellanTM Data Analysis V2. 30 Software (Tecan, Switzerland) was used.

### 2.5. Sulforhodamine B Assay (SRB-Assay)

The SRB assay was developed in 1990 and became a widely used application to measure apoptosis and cytotoxicity [26]. The method is based on stoichiometric binding of the dye to proteins under acidic pH. After a subsequent elution of the dye under basic conditions, a relative cell number compared to the negative control can be determined by absorbance measurement. To semi-quantify the cellular protein contents representing the approximate cell number [27], 1 × 10^4^ Cells/well were cultivated for three days in growth medium in 24-well plates containing DMEM-LG supplemented with 10% FCS and 1% P/S and incubated at 37 °C and 5% CO_2_. Differentiation was performed for 7 and 14 days, as described in Section 2.3. The cells were fixed with 4% paraformaldehyde for 20 min at room temperature. After washing two times for 5 min with deionized water, the cells were stained with 0.4% sulforhodamine B (SRB, Sigma-Aldrich, Taufkirchen, Germany) solution in 1% acetic acid for 10 min. The unbound color was removed via 4–5 washing steps for 5 min each with 1% acetic acid. The bound SRB was extracted with 500 µL of 10 mM unbuffered TRIS solution pH 10–10.5 for 30 min by gentle shaking, and 100 µL of extract was measured with a Tecan Sunrise absorbance microplate reader at an absorbance of 565 nm. The result is an approximate relative cell count based on the amount of protein.

### 2.6. MTT Assay

The 3-4,5-Dimethylthiazol-2yl-2,5-diphenyltetrazoliumbromid, MTT assay (Sigma-Aldrich, Taufkirchen, Germany) measures the level of tetrazolium reduction by Nicotinamide adenine dinucleotide phosphate-dependent cellular oxidoreductase exhibiting cell viability. The cells of all experimental groups were seeded at a density of 1 × 10^4^ Cells/well for three days in a growth medium in 24-well plates containing 10% FCS and 1% P/S in DMEM-LG. The cells were treated for 14 days with 3–4-day medium changes with each adipogenic additive alone in DMEM-LG to show which of the reagents used had a toxic effect. For the MTT assay, the medium was replaced by 300 μL of growth medium with 0.5 mg/mL MTT per well. The cells were incubated for 2 h under 37 °C and 5% CO_2_. The solution was discarded, and the cells were incubated with 200 μL of dimethylsulfoxide per well in triplicate on a shaker for 10 min at room temperature. A volume of 200 μL of each condition was transferred in triplicate into a 96-well plate. The absorbance was detected at 570 nm using a microplate reader equipped with Magellan TM Data Analysis V2.30 Software (Tecan, Switzerland).

### 2.7. Propidium Iodide Assay

In order to measure whether the individual adipogenic compounds induce cell apoptosis, a propidium iodide (PI) assay was employed [28]. PI is a red DNA-binding fluorescent dye that can only penetrate through the cell membrane of dead cells. Briefly, 1 x 10^4^ cells per well were seeded in a 24-well plate containing the growth medium for 3 days. The cells were incubated for 14 days with a growth medium supplemented with a single adipogenic agent each, such as IBMX, INDO, IBMX/INDO, DXM, ITS, and 1, 5, and 10 µM rosiglitazone. The medium change was performed every 3 to 4 days. The medium was discarded, and the cells were washed twice for 5 min with DMEM free of phenol red and FCS. The cells were incubated for 5 min in the dark with 10 μg/mL PI diluted in DMEM free of phenol red and FCS and washed three times for 5 min in DMEM free of phenol red and FCS. Five random microscopic fields per well for each condition (*n* = 3) were photographed using a fluorescence microscope equipped with AxioVision Rel. 4.8 software (Carl Zeiss Microscopy GmbH, Jena, Germany). The number of PI-positive cells per microscopic field was counted using ImageJ bundled with 64-bit Java 8 software.

### 2.8. RNA Preparation and Quantitative Real-Time RT-PCR

Adipogenic-induced cells were lysed at day 14 post-induction, and the total RNA was isolated using the GenElute Mammalian Total RNA Miniprep Kit (Sigma-Aldrich, Taufkirchen, Germany) according to the manufacturer’s instructions. First, 810 ng of total RNA was digested with 1.2 units of recombinant DNase I (Sigma-Aldrich, Taufkirchen, Germany) at 37 °C for 30 min to remove genomic DNA. The reverse transcribed cDNA was generated using MultiScribe™ Reverse Transcriptase (Thermo Fisher Scientific, Dreieich, Germany) and Random Hexamer Primer N6 (Genaxxon, Ulm, Germany), according to the manufacturer’s instructions. Real-Time Polymerase Chain Reaction (PCR) was performed with a CFX96 Touch Real-Time PCR Detection System (Biorad, Feldkirchen, Germany) using GoTaq^®^ qPCR Master Mix (Promega, Walldorf, Germany); the primers are shown in Table 1. Each experimental group was analyzed in triplicate, and 18S served as an endogenous housekeeper for all species. The data were analyzed using the gene expression equation Ct (2^−ΔΔCt^).

### 2.9. Statistical Analysis

Profiling of the collected data from three independent experimental setups (*n* = 3) was performed to assess the adipogenic differentiation protocols of ASCs, including ORO histologic staining, morphometric analysis of adipocytes, and quantification of the relative adipogenic markers, including Fatty acid binding proteins-4 (*FABP-4*) and *Leptin* expression. For the semi-quantitative analysis of cell numbers using SRB, morphometric measurements of adipocytes size (µm^2^) using ImageJ, quantification of the relative adipogenic markers *FABP-4* and *Leptin* expression, assessment of various adipogenic protocols at day 14, the percentage of normalized MTT absorbance measurement to the control (BM) value, and the counting of PI-positive cells, one-way ANOVA was carried out. To evaluate the effect of the adipogenic medium including IBMX, Rosi 1, and Rosi 5 compared with BM at different time points (day 7 vs. day 14) on the capacity of the adipogenic differentiation of ASCs by using semi-quantification of ORO staining, two-way ANOVA was performed. Multiple comparisons were tested using Tukey’s and Dunnett’s post hoc tests. All the data are presented as the mean ± SEM, and a *p*-value of ≤0.05 was determined to indicate significance. The analyses were performed using Graph Pad Prism 7.0 software (La Jolla, CA, USA).

## 3. Results

### 3.1. Morphological Evaluation of the Adipogenic Differentiation Capacity of ASCs in Various Species

**Figure 1:**
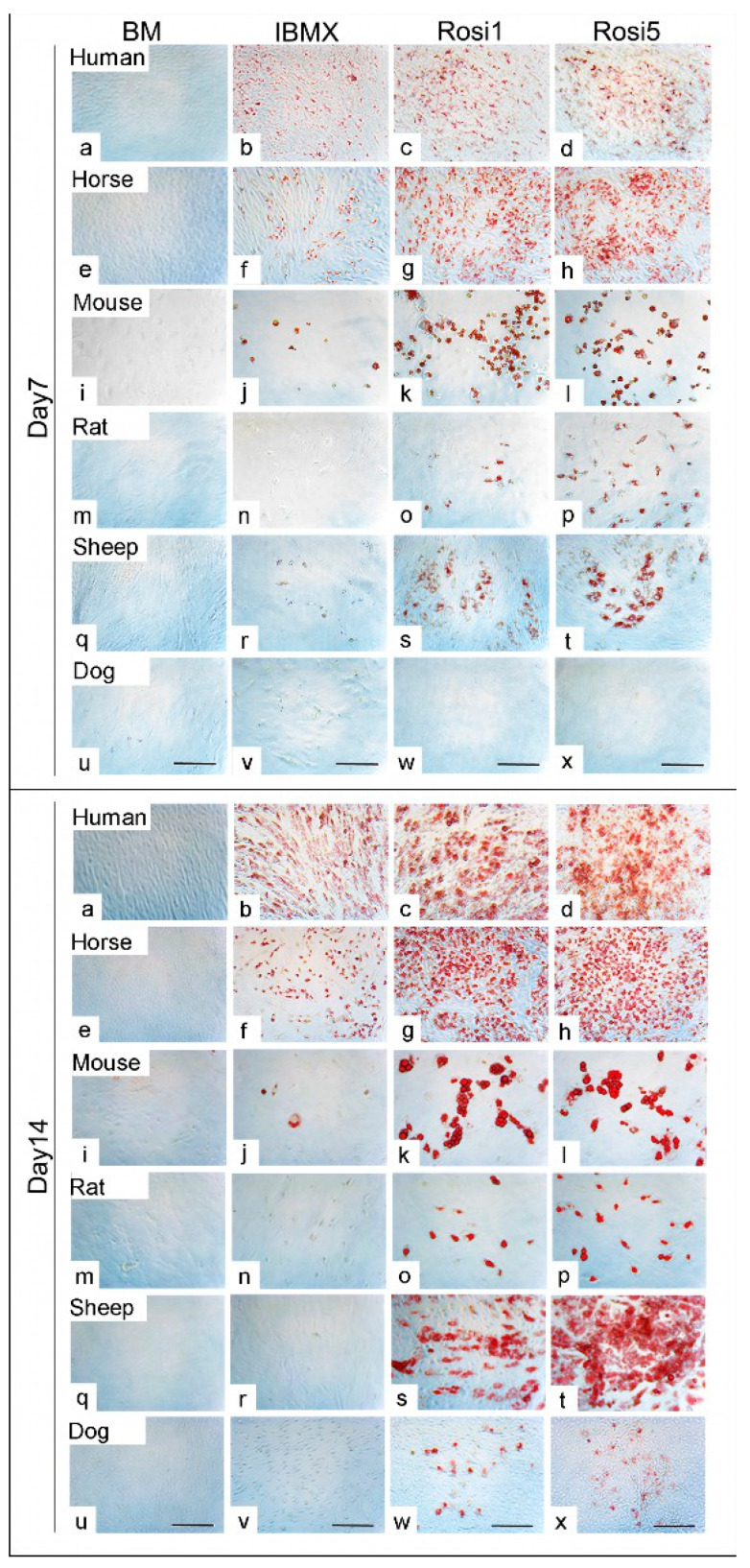
Morphological evaluation of the adipogenic differentiation capacity of adipose tissue-derived mesenchymal stem cells (ASCs) in various species (**a**–**x**) Histological staining of ASCs following adipogenic differentiation in 3-isobutyl-1-methylxantine-based medium (IBMX), 1 µM rosiglitazone (Rosi 1), and 5 µM rosiglitazone at day 7 and day 14 using Oil Red O (red) in human, horse, mouse, rat, sheep, and dog cells. Non-induced cells in basal medium (BM) served as negative controls. (**a**–**t**) Cells show evidence of adipogenic differentiation at day 7 in the presence of Rosi 1 and Rosi 5 compared with IBMX medium for human, horse, mouse, rat, and sheep cells. (**u**–**x**) No oil droplets in dog cells after differentiation at day 7. (**a**–**x**) Cells demonstrate promoted adipocytes formation at day 14 under Rosi 1- and Rosi 5-based conditions compared with IBMX medium and BM. There was a marked reduction in adipocyte formation in the presence of IBMX protocol at both time points. Scale bar = 100 µM.

The protocols currently used to induce adipogenic differentiation of MSCs need optimization in order to be valid for stem cells of various mammalian species, including horses, sheep, dogs, murines, and humans. In this study, we compare an old, commonly used protocol with a new protocol developed in our laboratory, in which IBMX and INDO have been replaced by certain concentrations of rosiglitazone. Morphological examination of differentiated cells showed the marked formation of fat droplets when either Rosi 1 or Rosi 5 was used compared to the standard protocol using IBMX medium. Already after the seventh day, a stronger differentiation could be observed in the rosiglitazone-induced cells in comparison to the IBMX-induced cells for all species except for the dog cells (Figure 1a–t). At day 7, no evidence of oil droplets could be detected following adipogenic induction in dog cells, indicating varying differentiation capacities of the cells between different species (Figure 1u–x). The effect was even more pronounced after 14 days of differentiation in terms of a marked increase in fat droplet formation in the presence of Rosi 1 and Rosi 5 inductions for human, horse, mouse, rat, and sheep cells compared with the IBMX condition (Figure 1a–t), although the standard IBMX protocol induced strong cell adipogenesis in horses and humans. However, adipogenesis using the same protocol did not work in mouse, rat, sheep, and dog cells by day 14. This is clearly demonstrated by histological staining with ORO staining (in Figure 1, compare b, f with j, n, r, v). In dog cells, the ability to form fat droplets was lower even when the Rosi medium was provided compared to all other species studied on day 7 (Figure 1u–x) and day 14 (Figure 1u–x).

### 3.2. Assessment of Cell Protein Contents Indicative for Cell Number after Adipogenic Induction

**Figure 2:**
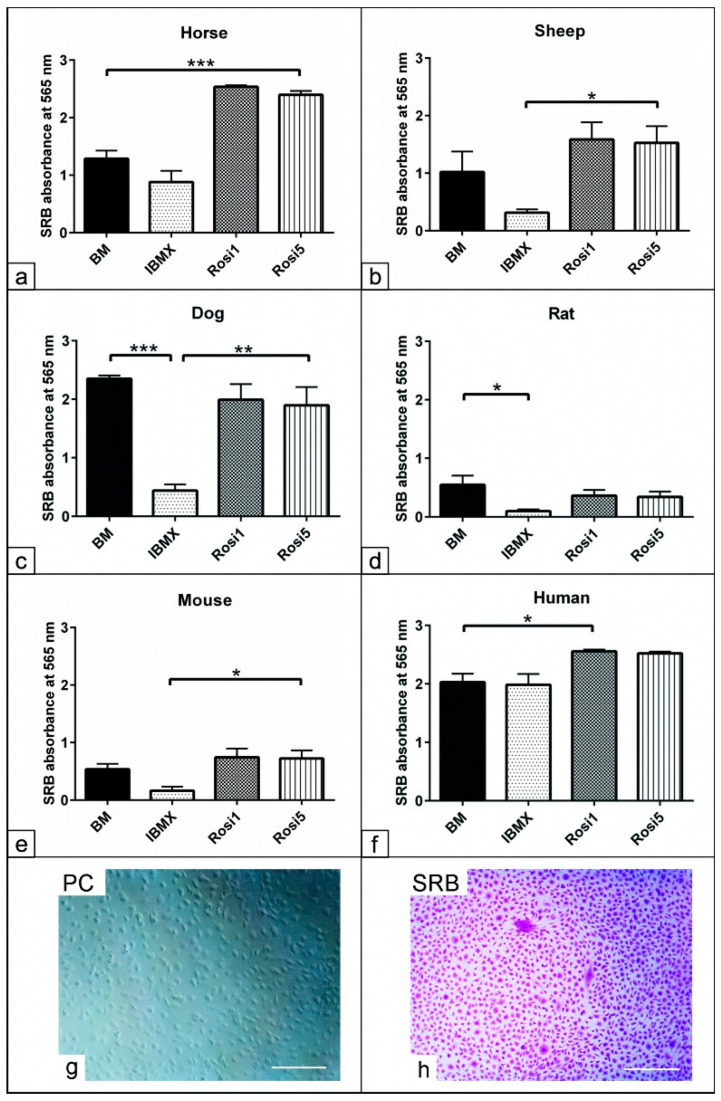
Assessment of cell protein contents indicative of cell number after adipogenic induction. (**a**–**h**) Semi-quantitative measurement of total protein content using SRB assay represents the cell numbers after day 14 in the presence of IBMX, 1 µM rosiglitazone (Rosi 1), and 5 µM rosiglitazone adipogenic medium. Non-induced cells in basal medium (BM) served as negative controls. Evaluation of the total protein content reveals increases in cell numbers in the presence of Rosi 1 and Rosi 5 compared with IBMX and BM in horse- (**a**), sheep- (**b**), mouse- (**e**), and human- (**f**) derived cells. Note the marked reduction in the cell numbers in the IBMX condition for all species except for human cells. Semi-quantitative analysis of total protein content using sulforhodamine B (SRB). (**g**) Phase contrast for ASCs of dog in growth medium at 14 days. (**h**) The cells were fixed with 4% paraformaldehyde for 20 min, and then were stained with 0.4% SRB solution (Violet) in 1% acetic acid for 10 min. All data are presented as the mean ± SEM. * = *p* < 0.05, ** = *p* < 0.01, *** = *p* < 0.001. Scale bar = 100 µM.

Morphological examination of the ASCs showed differences after cultivation in the different media compositions, resulting in adipogenic differentiation. To measure the cell viability and proliferation after using different media, the SRB assay was performed to indicate the protein content of the cells (Figure 2g,h). The data analysis showed that the cell numbers increased in the presence of the Rosi 1- and Rosi 5-based protocols in horse cells (*p* < 0.001) compared to cells cultivated in both BM and IBMX protocols (Figure 2a). Compared to the IBMX medium, adipogenically induced cells in the presence of the Rosi medium showed higher cell protein contents in sheep (*p* < 0.05), dog (*p* < 0.01), murine (*p* < 0.05), and human cells (*p* < 0.05, Figure 2b–f). A significant reduction in the cell protein levels could also be detected in the presence of IBMX compared to the non-induced cells in BM. These data indicate the effect of various components on the cell numbers during the course of adipogenic differentiation.

### 3.3. Evaluation of the Fat Content following Adipogenic Induction

**Figure 3:**
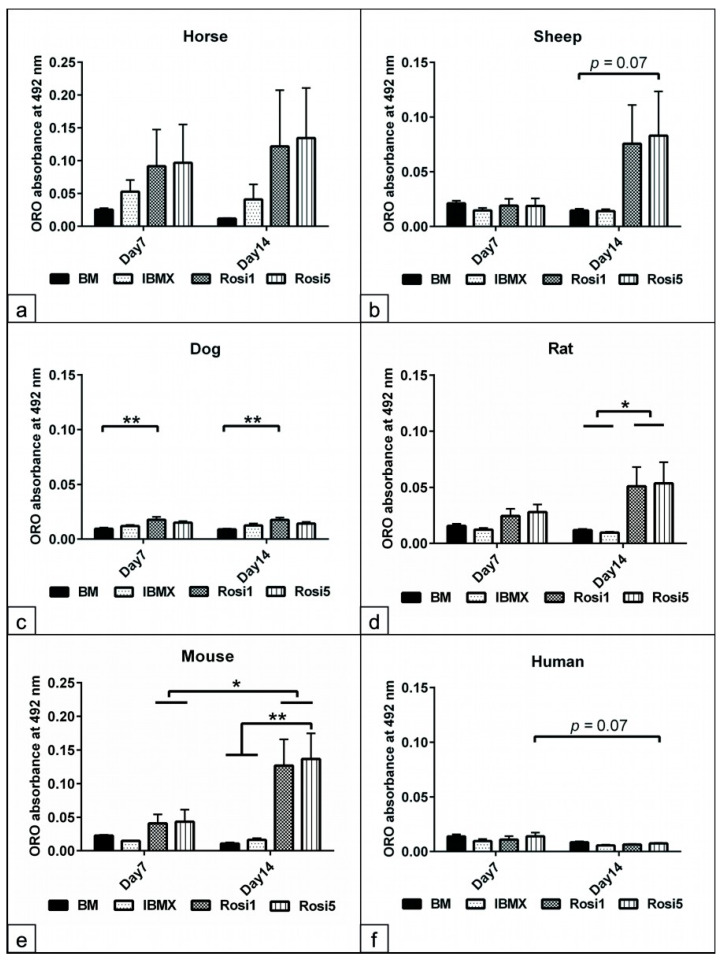
Evaluation of the fat content following adipogenic induction. (**a**–**f**) Semi-quantitative measurements of fat formation within adipocytes at day 7 and day 14 of adipogenic induction in the presence of IBMX, 1 µM rosiglitazone (Rosi 1), and 5 µM rosiglitazone adipogenic medium. Non-induced cells in basal medium (BM) served as negative controls. Oil Red O (ORO) stained cells were incubated with isopropanol for 30 min at RT with mild shaking in order to dissolve all bound stains. The acquired solution was loaded in 96-well plates (*n* = 4 per experimental group). The absorbance was measured in triplicate at 492 nm. Semi-quantitative analysis of the fat content in horses (**a**), sheep (**b**), dogs (**c**), rats (**d**), mice (**e**), and humans (**f**). Marked increases in cell adipogenesis up to day 14 when Rosi 1 and Rosi 5 were supplemented compared with IBMX and BM conditions. All data are presented as the mean ± SEM. * = *p* < 0.05, ** = *p* < 0.01.

In order to demonstrate that Rosi-modified protocols improve the adipogenic differentiation capacity of ASCs, a semi-quantitative analysis of the fat content in differentiated cells was performed on day 14. Although the fat content increased significantly after adipogenic induction on day 14 in horse and sheep cells, further analysis revealed only a tendency (*p* = 0.07) to an increase in the fat content compared to the IBMX protocol (Figure 3a,b,f). In addition, our data showed a significant increase in the fat content of dog (*p* < 0.01) and mouse (*p* < 0.01) cells at both day 7 and day 14 compared to cells cultivated in IBMX and BM media (Figure 3c,e). Rat cells differentiated with Rosi 1 and Rosi 5 showed a significant increase in fat content (*p* < 0.05) only at day 14 compared to the BM- and IBMX-induced cells (Figure 3d). We found that the fat content in mouse cells was dependent on the Rosi concentration used, i.e., the fat content with Rosi 5 was higher than that with Rosi 1 at day 14 after induction.

### 3.4. Morphometric Analysis of the Size of Adipocytes Following Adipogenic Induction

**Figure 4:**
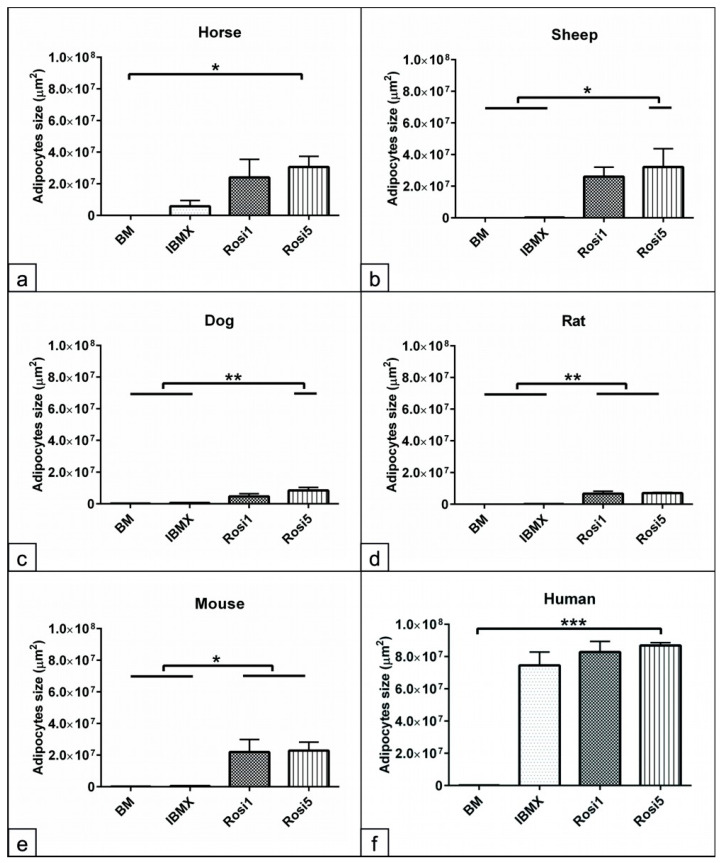
Morphometric analysis of the size of adipocytes following adipogenic induction. (**a**–**f**) Morphometric measurement of the size of adipocytes in horses (**a**), sheep (**b**), dogs (**c**), rats (**d**), mice (**e**), and humans (**f**) following 14 days in adipogenic induction media including IBMX, 1 µM rosiglitazone (Rosi 1), and 5 µM rosiglitazone adipogenic medium was performed using ImageJ bundled with 64-bit Java 8 software. Five random microscopic fields of ORO-stained cells representing the cultivation plate were photographed (*n* = 4 per experimental group). The size of all adipocytes within every image was measured (µm^2^). Non-induced cells in basal medium (BM) served as negative controls. Data analysis demonstrates increases in the size of adipocytes in the Rosi 1 and Rosi 5 conditions compared with the IBMX and BM conditions. Note the human cells show no significant differences in the size of adipocytes between IBMX and Rosi conditions. All data are presented as the mean ± SEM. * = *p* < 0.05, ** = *p* < 0.01 and *** = *p* < 0.001.

Having demonstrated that various differentiation protocols relatively affect the cell protein and fat contents of the adipogenic committed cells, as a next step using morphometric parameters, it was analyzed whether various adipogenic inducers altered the size of the adipocytes. Morphometric analyses at day 14 revealed a significant increase in the size of adipocytes in the Rosi 5-induced cells from horses (*p* < 0.05), sheep (*p* < 0.05), and dogs (*p* < 0.01) compared to BM or IBMX cultivated cells (Figure 4a–c). Data analysis showed that both Rosi 1 and Rosi 5 significantly increased the adipocyte size in the rat- (*p* < 0.01) and mouse (*p* < 0.05)-induced cells compared with the corresponding cells in IBMX and BM (Figure 4d,e). The results from human cells showed a significant increase in adipocyte size (*p* < 0.001) for IBMX, as well as Rosi1 and Rosi 5 compared to the non-induced cells in BM (Figure 4f). In summary, these data show that selection and optimization of the differentiation protocol can improve the capacity of ASCs and that the differentiation potential varies greatly between species under similar induction conditions.

### 3.5. Quantification of the Relevant Adipogenic Differentiation Marker Expression

**Figure 5:**
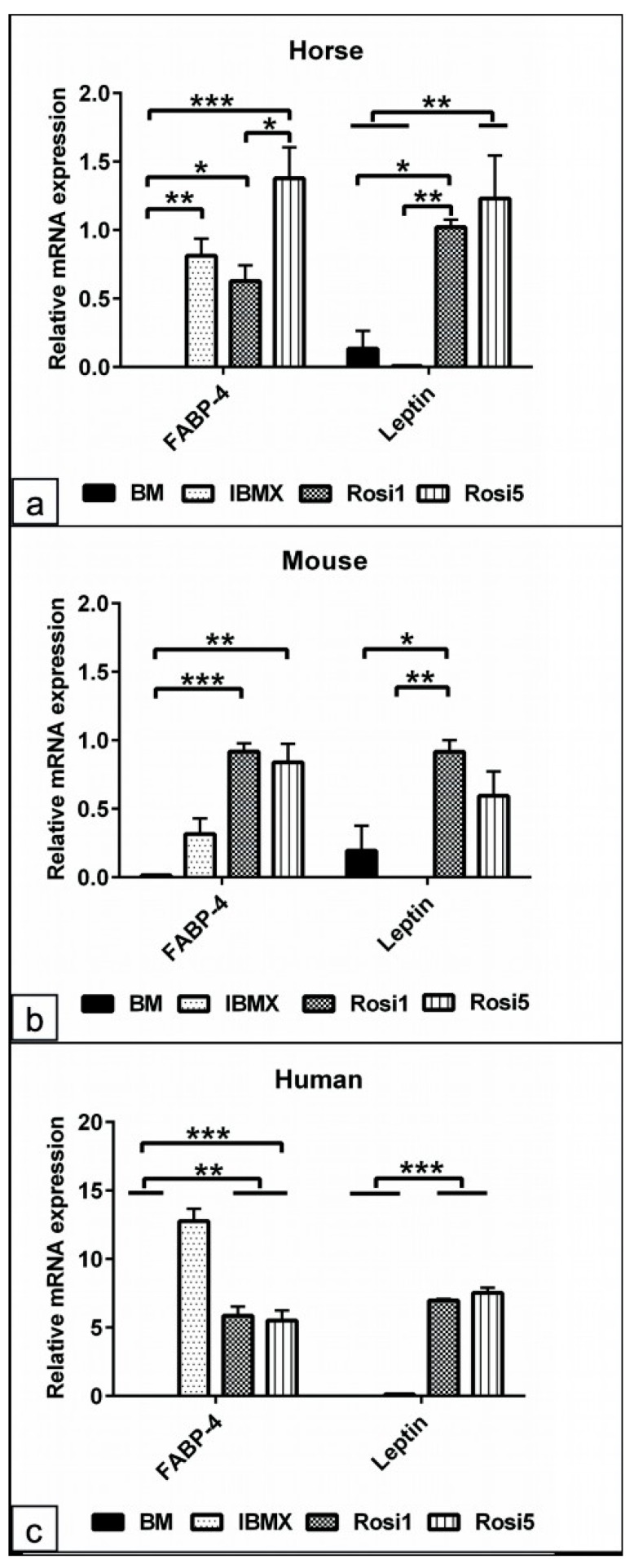
Quantification of the relevant adipogenic differentiation marker expression. (**a**–**c**) Quantitative RT-PCR of cells lysates from horse (**a**), mouse (**b**), and human (**c**) cells following 14 days of adipogenic induction in IBMX, 1 µM rosiglitazone (Rosi 1), and 5 µM rosiglitazone medium. For all experimental groups, 1 µg of the harvested RNA (*n* = 4) was reverse transcribed. By using the relevant primers, the adipogenic markers, fatty acid binding proteins-4 *(FABP-4),* and *Leptin* expression were measured. The data analysis reveals the upregulation of *FABP-4* expression in the Rosi 1-, Rosi 5-, and IBMX-based conditions compared with BM in all species. Note the marked inhibition of *Leptin* expression with IBMX condition in horse-, mouse-, and human-induced cells. All data are presented as the mean ± SEM. * = *p* < 0.05, ** = *p* < 0.01, *** = *p* < 0.001.

To investigate the effect of the different adipogenic protocols, the quantification of gene expression for markers specific for adipogenic differentiation, such as *FABP-4* and *Leptin,* was performed on day 14 in cells of all species (Figure 5 and Figure S2). Data analysis of the horse cells showed a higher *FABP-4* expression in all experimental groups, including IBMX (*p* < 0.01), Rosi 1 (*p* < 0.05), and Rosi 5 (*p* < 0.001), compared to the non-induced cells in BM, with the highest expression induced when the Rosi 5 condition was applied. *Leptin* was strongly upregulated in horse cells with both Rosi 1 and Rosi 5 (*p* < 0.01) compared to the corresponding IBMX and BM cells (Figure 5a). Analysis of rat and mouse cells showed that the expression of *FABP-4* was slightly elevated with IBMX but was not significant. However, a significant increase in *FABP-4* expression was observed with Rosi 1 (*p* < 0.05 and *p* < 0.001) and Rosi 5 (*p* < 0.05 and *p* < 0.01) compared with BM and IBMX. *Leptin* expression was only significantly increased with Rosi 1 (*p* < 0.05 and *p* < 0.01) compared to BM and IBMX, indicating that IBMX induction inhibited *Leptin* expression in rat and mouse cells (Figure 5b and Figure S2e,f). In human cells, increased *FABP-4* expression was observed with all protocols used, including IBMX (*p* < 0.001), Rosi 1 (*p* < 0.01), and Rosi 5 (*p* < 0.01), compared to non-induced cells. Surprisingly, the IBMX-based protocol induced a higher *FABP-4* expression than the Rosi-based media. In contrast, *Leptin* expression was higher in the presence of Rosi 1 and Rosi 5 (*p* < 0.001) compared to IBMX and BM cells (Figure 5c).

The sheep cells showed almost similar results as the horse cells, namely *FABP-4* was significantly higher expressed with Rosi 5 (*p* < 0.01) and Rosi 1 (*p* < 0.05) compared to IBMX and BM, and *Leptin* was upregulated with Rosi 1 (*p* < 0.05) and Rosi 5 (< 0.01), while no expression was seen in IBMX medium cultivated cells (Figure S2a,b). In dog cells, an upregulated expression of *FABP-4* (*p* < 0.05) and *Leptin* (*p* < 0.05 and *p* < 0.01) was detected with IBMX, Rosi 1, and Rosi 5, respectively, compared to the non-induced cells in BM (Figure S2c,d).

### 3.6. Assessment of the Common Adipogenic Inducers on ASCs Viability from Dog and Horse Cells

**Figure 6:**
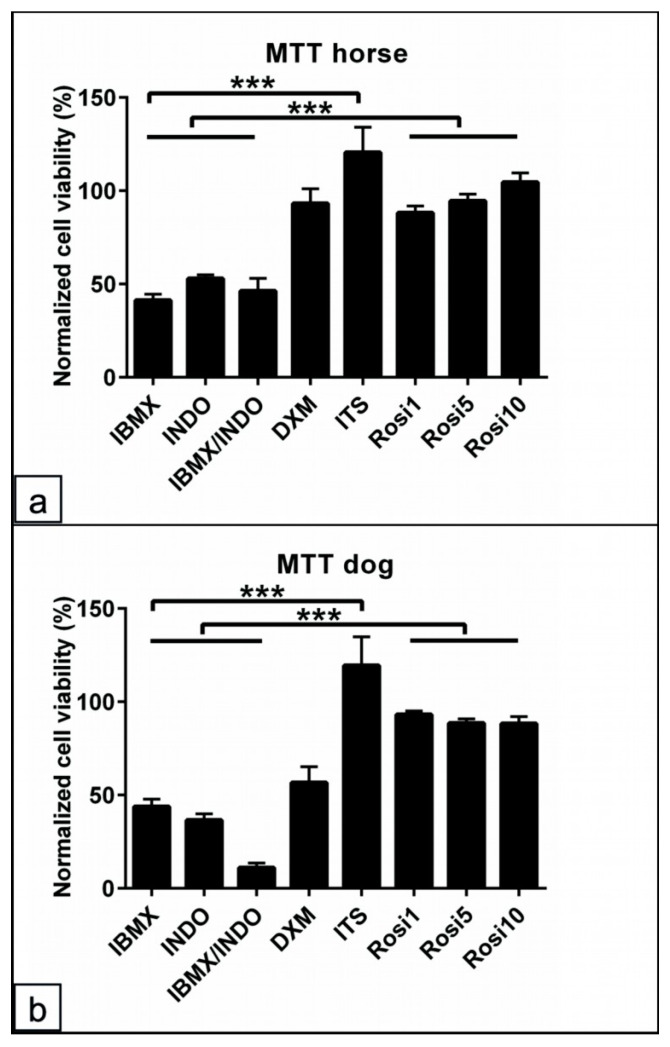
Assessment of the common adipogenic inducers on ASCs viability from dog and horse cells. (**a**,**b**) Percentage of normalized MTT absorbance to the control values for horse (**a**) and dog (**b**) cells following 48 h in various adipogenic inducers including IBMX, indomethacin (INDO), IBMX/INDO, dexamethasone (DXM), insulin–transferrinselenium (ITS), 1 µM rosiglitazone (Rosi 1), 5 µM rosiglitazone (Rosi 5), and 10 µM rosiglitazone (Rosi 10). Non-induced cells in basal medium (BM) served as the negative controls. The absorbance was examined at wavelength 570 nm to semi-quantify the cell viability. Analysis of the cell viability shows preserved cell viability for horse and dog cells in the presence of ITS-, Rosi 1-, Rosi 5-, and Rosi 10-supplemented medium compared with BM. A marked reduction in the cell viability in the conditions of IBMX, INDO, or in a combination of both can be detected. All data are presented as the mean ± SEM. *** = *p* < 0.001.

To show the effects of single components of the adipogenic medium, dog and horse cells were cultivated for 14 days in the BM with one supplement added successively. The cells in BM served as the negative control. The MTT assay was used to evaluate the cell viability, and PI staining was used to detect cell apoptosis/necrosis. The results of the normalized MTT data to the control values showed an up to 50% reduction in the cell viability with IBMX, INDO, and IBMX/INDO (*p* < 0.001) for horse and dog cells (Figure 6a,b). In contrast, the addition of ITS promoted the cell viability for the horse (*p* < 0.001) and dog (*p* < 0.001) cells and may overcome the toxic effect of other components, as mentioned above. Similarly, Rosi 1, Rosi 5, and Rosi 10 showed increased cell viability compared to IBMX, INDO, and IBMX/INDO (*p* < 0.001) for the cells of both species (Figure 6a,b).

**Figure 7:**
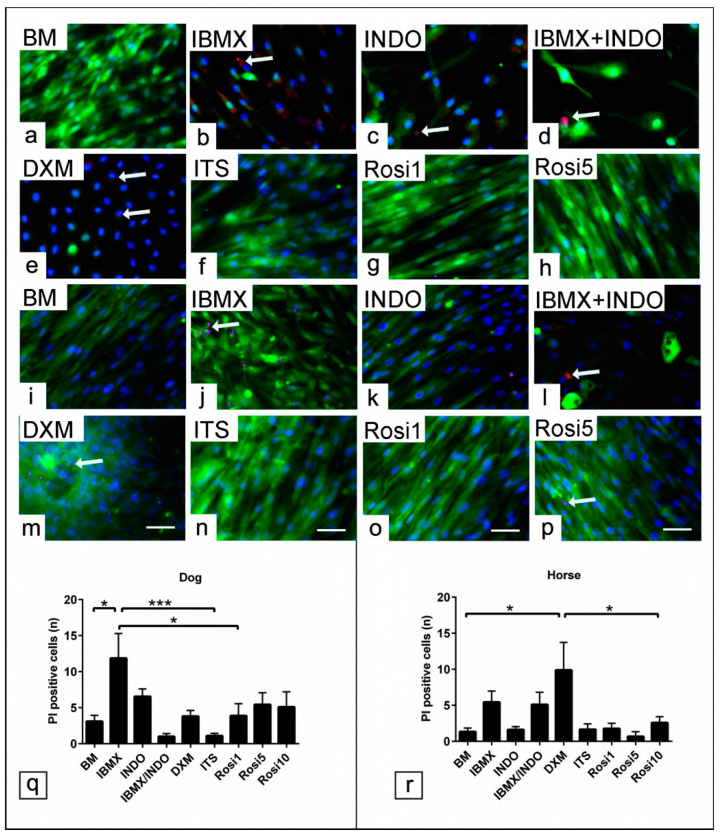
Effect of common adipogenic inducers on the cytotoxicity of ASCs. ASCs’ cultivation in various adipogenic inducers including IBMX, indomethacin (INDO), IBMX/INDO, dexamethasone (DXM), insulin–transferrin–selenium (ITS), 1 µM rosiglitazone (Rosi 1), 5 µM rosiglitazone (Rosi 5), and 10 µM rosiglitazone (Rosi 10) for 48 h in growth medium. Cell apoptotic elements tracking using propidium Iodide (PI) assay for viable cells. (**a**–**p**) Representative images show PI-positive cells (red) in dog (**a**–**h**) and horse cells (**i**–**p**). (**q**) Average number of PI-positive cells for dog cells (*n* = 3). (**r**) Average number of PI positive cells for horse cells (*n* = 3). Cells are counterstained with phalloidin (green). The analysis showed increases in cell apoptosis (arrow) when IBMX, INDO, IBMX/INDO, and DXM were added to the induction medium. Scale bar = 20 µm. All data are presented as the mean ± SEM. * = *p* < 0.05 and *** = *p* < 0.001.

PI staining showed a marked increase in apoptotic/necrotic cells in the presence of IBMX, INDO, and DXM in dog and horse cells (Figure 7a–p). The number of PI-positive cells in five random microscopic fields per experimental condition (*n* = 3) was counted. Analysis revealed that the number of PI-positive cells was increased in the presence of IBMX (*p* < 0.05, *p* < 0.001, and *p* < 0.05, respectively) compared with cells in BM, ITS, and Rosi 1 in dogs (Figure 7q). Similarly, PI-positive cells increased in the presence of DXM, IBMX, and INDO (*p* < 0.05) compared to cells in BM, ITS, and Rosi medium for horse cells (Figure 7r). Taken together, the data clearly demonstrate that proper selection of adipogenic components not only can influence the cell proliferation and differentiation ability but also may preserve the cell population from apoptosis.

## 4. Discussion

It is well established that the balance between caloric intake and energy expenditure is the key modulator for adipogenesis. A high energy intake recruits MSCs’ commitment into adipocyte precursors that differentiate into adipocytes packed with fat droplets. Investigating the adipogenic differentiation of ASCs increases our understanding of the pathophysiology of metabolic syndromes, diabetes, and the consequence of obesity in veterinary medicine; see the review of [29]. In addition, evaluating the molecular regulation in the course of adipogenesis [30] by investigating ASCs of various mammalian species facilitates the assessment of drug activity on fat synthesis and fat metabolism under both physiological and pathological conditions [31,32]. The abundance of fat tissue and straightforward adipogenic differentiation of ASCs inspired stem cells to be an attractive model to examine the mechanism of adipogenesis. However, the discrepancies between the induction protocols and the absence of common evaluation criteria could impact the differentiation capacity of the stem cell pool, which requires special attention. ASCs’ isolation and characterization protocols were previously reported from our group for horse [23], dog [24], and rat cells [25].

In the present study, we compared the most commonly used protocol to induce adipogenic differentiation with an optimized rosiglitazone-based protocol not only to overcome the toxic effect of adipogenic inducers on cell viability but also to promote the adipogenic capacity of stem cells in a wide range of mammalian species. The experimental procedures were carried out on ASCs of matched passage (P1-4), age range, and sex from horses, sheep, dogs, murines, and humans in order to avoid the effect of cell senescence in higher passages, as well as to exclude the effect of sex and aging on stem cell performance. The results of histologic staining, morphometric measurements, and fat droplet quantification as a readout revealed that using Rosi 1- and Rosi 5-based medium increased the adipogenic capacity of ASCs up to day 14 in equine, ovine, canine, and murine cells compared to IBMX- and INDO-based induction. This could also be shown exemplarily as a proof-of-concept for promoted oil droplets’ formation indicative of adipogenic differentiation in the presence of rosiglitazone in cat and chicken cells compared to IBMX conditions (Figure S1b–g). Our results pointed out the efficiency of the PPARγ agonist rosiglitazone to promote adipogenic differentiation compared to the IBMX/INDO conditions. A large group of evidence reported that PPARγ is the master regulator of adipogenic differentiation, including the enhancer binding protein alpha (C/EBPα), in order to trigger the respective adipogenic gene expression [33]. In the same line, a study demonstrated that rosiglitazone treatment enhanced the adipogenic differentiation of canine ASCs. However, its effect can be depleted by adding the PPARγ antagonist GW9662, which results in a reduction in cell differentiation [34]. The effect of rosiglitazone has been reported in other cell types too. In a study using human muscle satellite cells, it was found that rosiglitazone promoted adipogenic differentiation and activated PPARγ expression, which gives a hint of the presence of adipocytes in the skeletal muscle, as can be shown for diabetes and obesity during the course of aging [35]. In the last few years, several studies have shown that standard protocol components such as DXM, IBMX, and INDO induce adipogenic differentiation via a mechanism including the activation of the glucocorticoid receptors and also by either targeting cAMP signaling or cyclooxygenases’ (COX-1 and COX-2) inhibition [36,37,38]. However, the present study showed a superior effect by adding rosiglitazone to the induction medium at a concentration range from 1 µM up to 10 µM. These results suggest that rosiglitazone, a member of thiazolidinedione’s family, could directly trigger PPARγ, the common coordinator for adipogenic differentiation [39]. In the same context, it has been found that PPARγ activation is sufficient and the key regulator to drive adipogenic differentiation in both in vivo and in vitro conditions. The mechanism of rosiglitazone is due to the direct targeting of the nuclear receptor PPARγ, which is necessary to trigger adipogenic differentiation [40]. In contrast, DXM stimulates the glucocorticoid receptor and C/EBPδ [38], which mostly modulate the intensity of lipid accumulation [41,42]. On the other hand, IBMX induces adipogenesis by activating the cAMP-dependent protein kinase and c/EBPβ signaling pathways [10].

Furthermore, it was reported that rosiglitazone treatment improves cell sensitivity to insulin without targeting the pancreatic beta cells. Thus, rosiglitazone plays an important role in both adipogenesis and glucose metabolism [43]. Our data reveal that a rosiglitazone concentration of up to 5 µM was suitable to promote adipogenesis without negatively affecting cell viability. In agreement with our results, it can be suggested that in most species, 5 µM rosiglitazone should be added to the standard adipogenic medium. In contrast, antagonizing PPARγ by using T0070907 and GW9662 treatments effectively inhibits triglyceride formation in 3T3-L1 and OP9 preadipocyte cells [42]. Along this line, rosiglitazone administration increases adipocyte trans- differentiation from white to brown fat tissue via a mechanism including enhanced mitochondrial biogenesis and oxidation of fatty acids [44,45].

Our data demonstrate a marked reduction in cell viability following the addition of IBMX, INDO, and combined IBMX together with INDO. This points out that although these compounds stimulate adipogenesis, they impair cell performance and induce cell cytotoxicity. In contrast, differentiation supplements, including ITS and rosiglitazone in various concentrations, maintain cell viability and increase the number of the differentiated cell population, stressing the suitability of using rosiglitazone for an adipogenic induction protocol by maintaining the cell population at the same time. The results showed an enhanced cell proliferation indicated by increases in the cell protein content. As a consequence, using a differentiation medium altered by the addition of rosiglitazone can be recommended in most species, including humans. In contrast, the protocol supplemented with IBMX/INDO showed increases in cell apoptosis, as shown by using the PI assay, which has clearly been demonstrated for canine and equine cells. The possible explanation would be that, on the one hand, the adipogenic precursors cultivated in a differentiation medium, including rosiglitazone, require a certain period to switch from the proliferation to the differentiation state. On the other hand, another explanation would be that treatment with rosiglitazone promotes the division of preadipocytes before they become mitotically inactive. Thus, a larger cell number would be available for terminal differentiation. In agreement with our hypothesis, it has been concluded that adipocytes might not be fully differentiated following adipogenic induction and that these cells maintain a proliferative ability, as has similarly been reported for cardiomyocytes [46,47]. Furthermore, it was documented that adipogenic induction using insulin growth factor1 (IGF1), glucocorticoid, and cAMP promotes preadipocytes to re-enter a cyclic division termed mitotic clonal expansion, which is essential before adipogenic differentiation [48,49,50]. These data suggest that although IBMX-based conditions drive adipogenesis to some extent, they reduce the proliferative ability of the stem cell population due to an increase in apoptotic side effects. In agreement with our data, one study revealed that although INDO promoted adipogenesis by targeting PPARγ expression, it caused cytotoxicity following adipogenic induction of canine ASCs [34]. The present data point out that IBMX, a cyclic AMP activator, impairs cell proliferation and promotes apoptotic activities in the course of adipogenic differentiation. In accordance with the present data, another study examined the molecular interaction between cAMP and *Leptin* in breast cancer cells; it was concluded that cAMP has an antiproliferative activity by targeting cell cycle progression, and the elevation of cAMP potentiates *Leptin* to induce cell apoptosis [51]. Our data provide evidence that the apoptotic activity in the case of IBMX, INDO, and DXM conditions probably occurs due to the interference with cyclic progression, leading to a decrease in the number of preadipocytes, which subsequently lowers the differentiation capacity of cells. Along the same lines, it was found that the overexpression of the key cell cycle regulator p27 interrupts the cyclic progression towards the S-phase and thus prevents the terminal differentiation of preadipocytes [52]. The previous reports support our data in terms of the apoptotic activity of the common adipogenic inducers, in particular, IBMX, INDO, and DXM, which impact cell proliferation and differentiation.

The morphometric analysis points out that the induction medium supplemented with 5 µm of rosiglitazone increases the size of the adipocytes in equine, ovine, and canine cells compared to 1 µm rosiglitazone. In addition, all the rosiglitazone concentrations were able to increase the size of adipocytes in rat and mouse cells. Thus, our data not only emphasize the interspecies differences regarding the adipogenic differentiation potential under a similar induction but also highlight the sensitivity of murine cells to a lower concentration of rosiglitazone compared to other species. The data might be valuable regarding the validity of animal models for investigating diabetes treatment applications. Our interpretation was in agreement with a study evaluating the suitability of animal models for diabetes. The study concluded that based on the diagnosis parameters, including glucose and glycosylated hemoglobin (HbA1c), the data revealed the similarity between the rat animal model and human diabetes following rosiglitazone treatment [53]. In the increases in increases in the possibility of enhanced insulin sensitivity, as was also shown in a study investigating the effect of FK614 and pioglitazone, a thiazolidinedione PPARγ agonist in the Zucker fatty rat, an animal model for diabetes [54]. Data from our study revealed a common trend of increased adipocyte size in all adipogenic induction protocols for human cells, which may be caused by increases in lipid droplet formation followed by the fusion of these droplets. Surprisingly, the protocol, including IBMX, resulted in a higher adipogenic differentiation capacity in human and equine cells but not in cells of other species. A possible explanation for this would be that cells of human and equine origin might be more sensitive to the IBMX induction medium compared to cells of other species. Along the same lines, a recent report revealed that IBMX combined with rosiglitazone effectively improved the adipogenic differentiation capacity for canine ASCs differentiated for up to 21 days [34]. However, in the present study, we showed an efficient adipogenic induction with rosiglitazone while maintaining the cell population viability and minimizing the risk of cytotoxicity, as shown in the IBMX condition.

Furthermore, our data analysis revealed upregulation of *FABP-4* and *Leptin* expression when the rosiglitazone-based protocol was used, as shown for ovine and equine cells. In contrast, *Leptin* expression was not detected in the presence of the IBMX-based protocol in most species. The data point out a selective gene expression under different protocols, suggesting that *FABP-4* might be one of rosiglitazone’s downstream targets for promoting adipogenesis. In accordance with our data, it has been reported that PPARγ interacts with C/EBPα to regulate adipogenic gene expression [55,56]. Along the same lines, a previous study revealed enhanced PPARγ and C/EBPα expression under combined IBMX, DXM, insulin, and rosiglitazone induction in canine ASCs, which upregulates the adipogenic markers, including *FABP-4*, lipoprotein lipase (*LPL*), and *Leptin* expression [34]. According to our data, there is an upregulation of the adipogenic marker expression under rosiglitazone-based protocols. However, analyzing human cells cultivated under an IBMX-based protocol, an upregulated *FABP-4* but no upregulated *Leptin* expression can be detected, emphasizing a particular sensitivity of human cells to IBMX. In this context, it has previously been reported that there is a combined effect of PPARγ and C/EBP for the promotion of adipocyte differentiation. This is in line with a study that concluded that an induction of *Leptin* is important for maintaining energy homeostasis [57]. The present data show evidence of a modulatory effect of IBMX on *Leptin* expression. In this context, a similar study concluded that *Leptin* increases insulin secretion in mouse HIT-T15 pancreatic islet cells. This effect was depleted when 100 mM of IBMX was added, suggesting that the level of intracellular cyclic AMP controls the effect of *Leptin* on insulin production [58]. Herein, we could suggest that although IBMX drives adipogenic differentiation, it has a systemic negative effect on insulin metabolism.

## 5. Conclusions

The present study reports that rosiglitazone provides a potent PPARγ2 agonist to safely drive adipogenesis of ASCs in a wide range of animal species. We show that a rosiglitazone-modified protocol preserves cell proliferation and differentiation capacities and minimizes cell toxicity compared to IBMX-based conditions. Our data support previous studies, which documented preadipocyte division before terminal differentiation. Furthermore, we provide evidence that the widely used IBMX/INDO conditions increase the risk of apoptosis in the stem cell pool, thus impairing their differentiation potential. We observed that murine cells are sensitive to 1 µM rosiglitazone to trigger adipogenic markers; however, 5 µM of rosiglitazone is necessary to switch on adipogenic differentiation in other species, which emphasizes the interspecies differences in terms of cell response to both the induction protocols and the expression of relevant adipogenic cues. The selective gene expression under the influence of different protocols suggests that *FABP-4* might be one of rosiglitazone’s downstream targets to promote adipogenesis. Altogether, we provide evidence that rosiglitazone-modified protocols, in contrast to IBMX/INDO-based induction, are more suitable for the adipogenesis of ASCs and minimize cytotoxicity. The data presented here are an important report on differentiating ASCs in various animal species not only for further investigation of adipose tissue pathophysiology but also for stem cells’ reproducibility and therapeutic applications targeting obesity in veterinary medicine.

## Figures and Tables

**Table 1:** Primer sequences and accession numbers for RT-qPCR.

Species	Gene	Forward	Reverse	Size (bp)	Accession Number
Human	FABP-4	GTAGGTACCTGGAAACTTGTC	TCCCCATTCACACTGATGATC	127	NM_001442.2
	LEPTIN	GTGCCCATCCAAAAAGTCC	GGAGCCCAGGAATGAAGTC	136	NM_000230.2
Horse	FABP-4	GTAGGCACCTGGAAACTTGTC	CCCCATTCACACTGATGATC	127	XM_005613035.3
	LEPTIN	CGAAAAGTCCAGGATGACAC	AACCAGTGACCCTCTGTTTG	106	NM_001163980.1
Sheep	FABP-4	ATCAGTGTAAATGGGGATGTG	GACTTTCCTGTCATCTGGAGTGA	117	NM_001114667
	LEPTIN	ACCCCTGTACCGATTCCTG	GCGTGTGTGAGATGTCATTG	134	XM_004008038.3
Dog	FABP-4	ATCAGTGTAAACGGGGATGTG	GACTTTTCTGTCATCCGCAGTA	117	XM_845069.5
	LEPTIN	GTGCCAATCCGAAAAGTCC	GGAGCCCAGGAATGAAGTC	136	NM_001003070.1
Rat	FABP-4	GTGGGGACCTGGAAACTCGTC	TCCCCTTCTACGCTGATGATC	127	NM_053365.1
	LEPTIN	CAGGATGACACCAAAACCC	TGAAGCCCGGGAATGAAGTC	119	NM_013076.3
Mouse	FABP-4	CATCAGCGTAAATGGGGATTTG	CTTCCTGTCGTCTGCGGTGA	115	NM_024406.2
	LEPTIN	CAGGATGACACCAAAACCC	TGAAGCCCAGGAATGAAGTC	119	NM_008493.3
	18S	ATGCGGCGGCGTTATTCC	GCTATCAATCTGTCAATCCTGTCC	204	NR_145820.1

## Data Availability

The data presented in this study are available upon reasonable request from the corresponding author. The data are not publicly available due to privacy.

## Supplementary Materials

The following are available online at https://www.mdpi.com/article/10.3390/ani13203224/s1. Figure S1: (a) This schematic diagram illustrates the study design, including questions, hypothesis and aims, experimental flow, targeted objectives, and the main results. (b–g) Representative images of ASCs stained with Oil Red O (red) following adipogenic differentiation for cat and chicken. Figure S2: Quantification of the relevant adipogenic differentiation markers expression including FABP-4 and Leptin for sheep (a,b), dog (c,d), and rat (e,f). Table S1: Antibodies used for ASCs identification.

## Abbreviations

MSCs	Mesenchymal Stem Cells
ASCs BMSCs	Adipose tissue-derived mesenchymal stem cells Bone marrow-derived mesenchymal stem cells
BM	Basal medium
IBMX	3-isobutyl-1-methylxantine
ROSI	Rosiglitazone
INDO	Indomethacin
DXM	Dexamethasone
ITS	Insulin–transferrin–sodium selenite
cAMP	Cyclic adenosine monophosphate
cGMP	Guanosine 3′,5′-cyclic monophosphate
PDEs	Cyclic nucleotide phosphodiesterase
C/EBPβ	CCAAT enhancer binding protein
C/EBPδ	Transcription factor of the basic leucine zipper
COX1, 2	Cyclooxygenases
PPARγ	Peroxisome proliferator-activated receptor gamma
FABP-4	Fatty acid-binding protein-4
ORO	Oil Red O
SRB	Sulforhodamine B
MTT	(3-(4,5-Dimethylthiazol-2-yl)-2,5-Dimethyltetrazolium Bromide) cell viability assay
PI	Propidium iodide
