# Supplementary material for: Optimizing the Adipogenic Induction Protocol Using Rosiglitazone Improves the Physiological Parameters and Differentiation Capacity of Adipose Tissue-Derived Mesenchymal Stem Cells for Horses, Sheep, Dogs, Murines, and Humans
Source: Animals (Basel). 2023 Oct 15;13(20):3224. doi: 10.3390/ani13203224 (PMC10603751; doi:10.3390/ani13203224)
Supplement: Supplementary file 1 [file animals-13-03224-s001.zip › animals-2628377-supplementary.pdf]

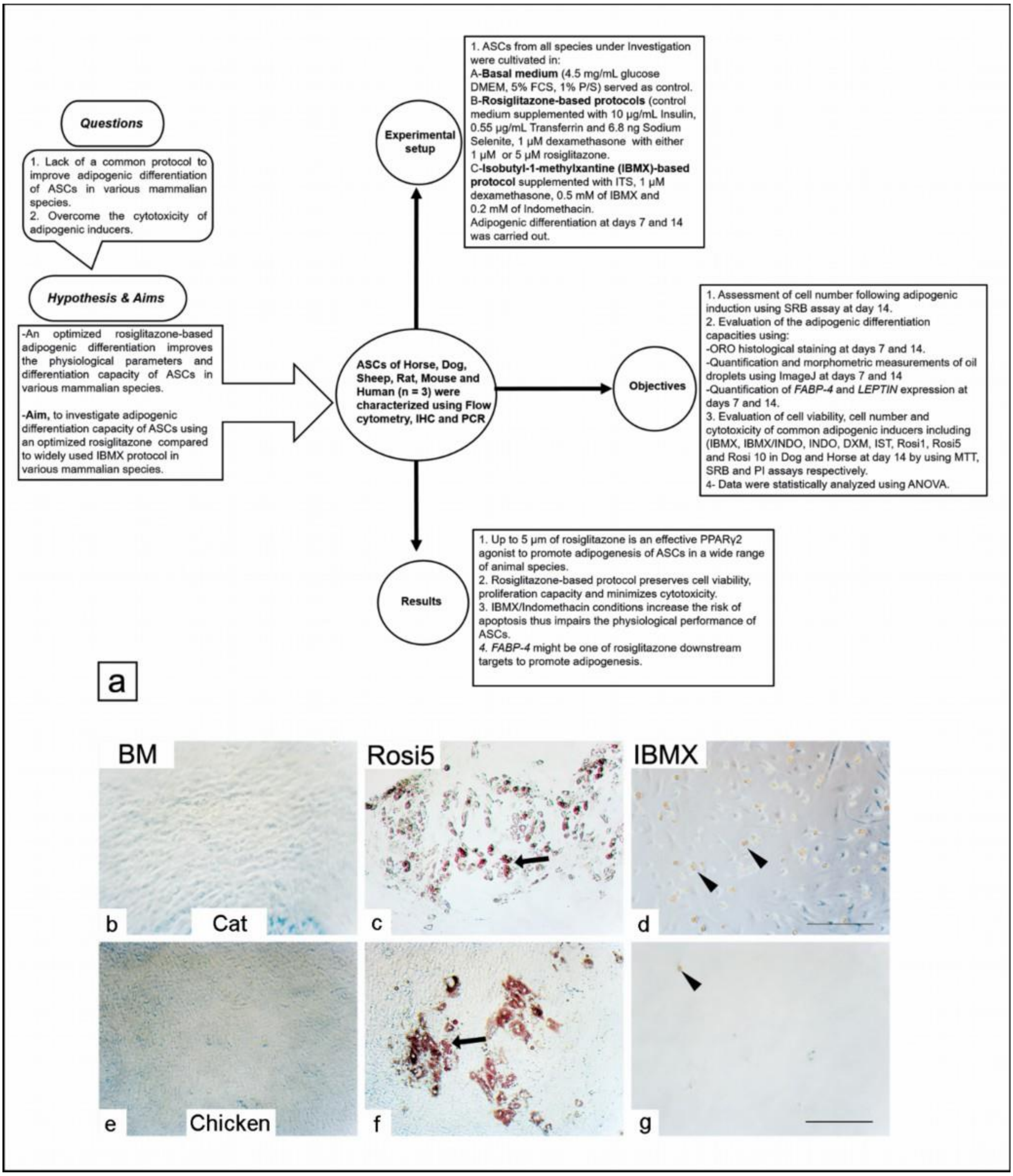

Figure S1: (a) This schematic diagram illustrates the study design including questions, hypothesis and aims, experimental flow, targeted objectives and the main results. (b–g) Representative images of 901 ASCs stained with Oil Red O (red) following adipogenic differentiation in 5 µM Rosiglitazone (Rosi 5) and 3-isobutyl-1-methylxanthine-based mediom (IBMX) up to day 14 post induction in cat (c, d) and chicken (f, g). Non-induced cells in basal medium (BM, b, e) served as negative controls. Evidence of promoted adipogenic differentiation as indicated 904 by red fat droplets (black arrow) under 5 µM of rosiglitazone compared to IBMX-based protocol. Note: increased detached round cells (arrow head) in IBMX condition for both species. Scale bar = 100 µM.

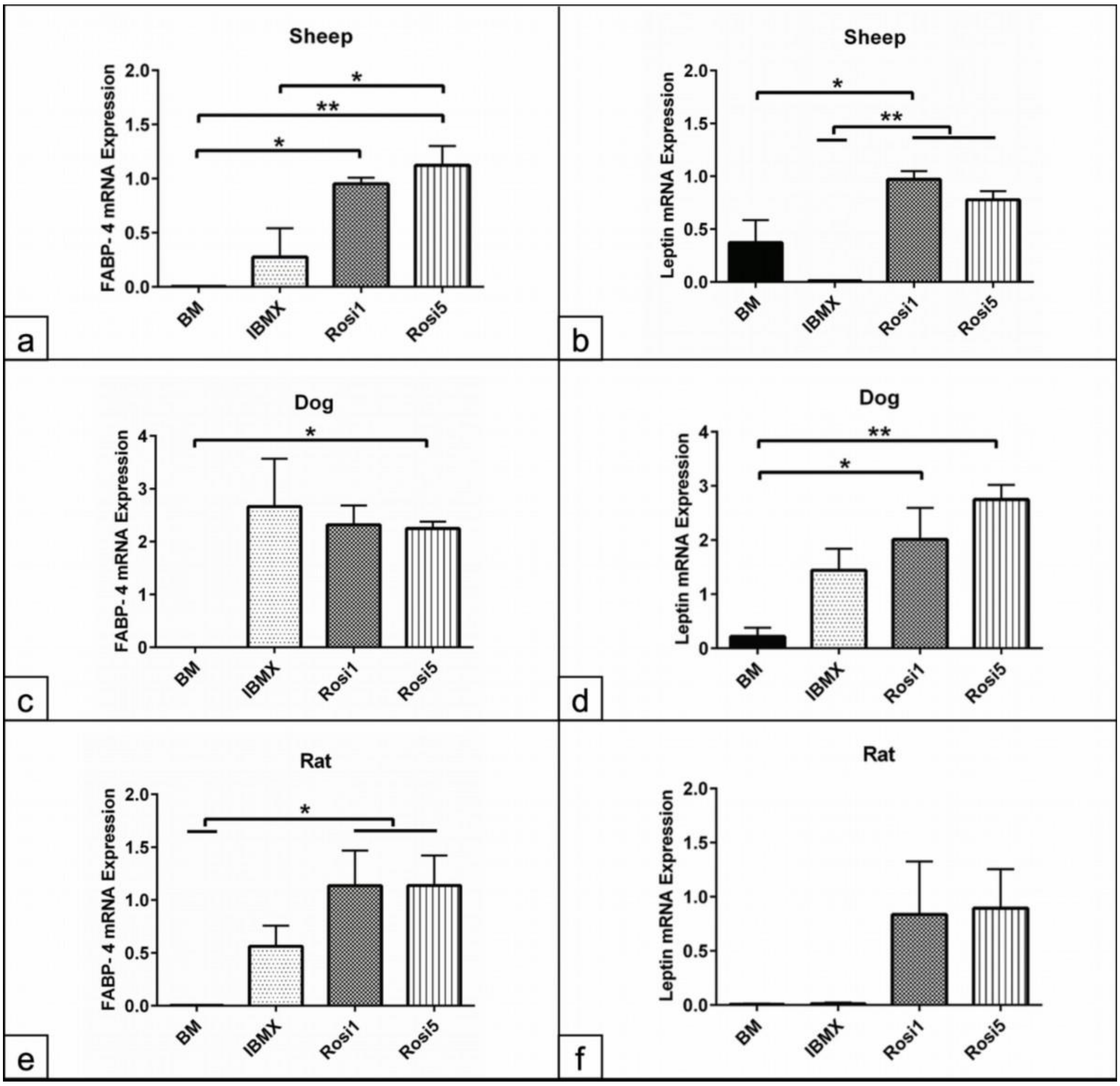

Figure S2. Quantification of the relevant adipogenic differentiation marker expression. (a–f) Quantitative RT-PCR of cells lysates from sheep (a, b), dog (c, d), rat (e, f) cells following 14 days of adipogenic induction in 3-isobutyl-1-methylxanthine-based medium (IBMX), 1 μM rosiglitazone (Rosi 1), 5 μM rosiglitazone medium. For all experimental groups, 1 μg of the harvested RNA (n = 4) was reverse transcribed. By using the relevant primers, the adipogenic markers; Fatty acid binding proteins-4 (*FABP-4*) and *Leptin* expression were measured. The data analysis reveals the upregulation of FABP-4 expression in the Rosi 1- and Rosi 5-, and IBMX-based conditions compared with BM in all species. All data presented as mean ± SEM. *= $p < 0.05$, **= $p < 0.01$.

Table S1: Antibodies used for ASCs identification

Positive-Marker	*Company*	*Host species*	*Target*	*Human*	*Dog*	*Horse*	*Sheep*	*Rat*	*Mouse*
CD29 # Ha2/5	BD Pharmingen	Hamster	Ra					+	+
CD29 # MEM101A	Biomol	Mouse	Human	+	+		+		
CD44 # IM7	Thermofisher	Rat	Mouse	+		+		+	+
CD44 # MEM-263	Novusbio	Mouse	COS-7 cells	+	+		+		
CD73 # 5F/B9	BD Pharmingen	Mouse	Rat					+	
CD90 # 5E10	Stemcell	Mouse	Human	+		+			
CD90 # YKIX337.217	Biorad	Rat	Dog		+				
CD90 # OX-7	Biorad	Mouse	Rat					+	+
CD90 # Thy1.2	Thermofisher	Rat	Mouse						
CD105 # SN6	Biorad	Mouse	Human	+		+			
MHC I # CVS22	Biorad	Mouse	Horse			+			
Negative-Marker									
CD45 # UHCL1	Thermofisher	Mouse	Human	+	+				
CD45 # OX-1	Biorad	Mouse	Rat					+	
CD45 # 30F11	Miltenyibiotec	Rat	Mouse						+
MHC II # CVS20	Biorad	Mouse	Horse	+	+	+			
CD11b # WT.5	BD Pharmingen	Mouse	Rat					+	
CD41/CD61 #CO.35E4	Biorad	Mouse	Sheep			+	+		

(+) positive signal
